# Vulnerability and adaptation to climate change in the Comoe River Basin (West Africa)

**DOI:** 10.1186/s40064-016-2491-z

**Published:** 2016-06-23

**Authors:** Wonnan Eugène Yéo, Bi Tié Albert Goula, Bernd Diekkrüger, Abel Afouda

**Affiliations:** GRP CC & Water Resources, University Abomey Calavi, Cotonou, Benin; Faculty of Science and Environment Management, University Nangui Abrogoua, Abidjan, Cote d’Ivoire; Department of Geography, University of Bonn, Bonn, Germany

**Keywords:** Climate change, Vulnerability, Water user, Adaptation strategy, Comoe River Basin

## Abstract

Climate change is impacting water users in many sectors: water supply, farming, industry, hydropower, fishing, housing, navigation and health. Existing situations, like population growth, movement of populations from rural to urban areas, poverty and pollution can aggravate the impacts of climate change. The aim of the study is to evaluate the vulnerability of different water user groups to climate change and define communities’ adaptation strategies in the Comoe River Basin. Information was collected on communities’ concerns and perception on changes in climate and potential adaptation measures and strategies. Results show that 95 % of the sample in the study communities had heard of it and are aware that climate change is occurring. They have been experiencing changes in economic activity and cropping pattern, reduced water level in rivers, crop failure, delay in cropping season, new pests and diseases, food insecurity, drop in income and decline in crop yield. Results also show that communities employ various adaptation strategies including crops diversification, substitution and calendar redefinition, agroforestry, borrowing from friends and money lenders and increasing fertilizer application.

## Background

CCPAN (2007) and Hope (2011) have defined climate change as any long-term significant regional change in measures of climate (such as temperature, precipitation, or wind patterns). Also, they argued that it is therefore any major long-term variation in the average weather that a given region experiences. They have stated that these variations must be statistically significant in measurements of either the mean state or variability of the climate for that region, whether due to natural factors or as a result of human activity. For them, climate change can, consequently, be regarded as a change of climate, which is attributed directly or indirectly to human activity, and which alters the composition of the global or regional atmosphere, in addition to natural climate variability over comparable time periods.

According to Fussel (2005) and weADAPT (2011), “vulnerability is a central concept in a variety of research contexts such as natural hazards and disaster management, ecology, public health, poverty and development, secure livelihoods and famine, sustainability science, land change, and climate impacts and adaptation”. Parry et al. (2007) have defined vulnerability as “the degree to which a system is susceptible to, or unable to cope with, adverse effects of climate change, including climate variability and extremes”. Vulnerability depends on exposure, sensitivity and adaptive capacity. Water users are vulnerable to changes in water resources depending on the level of socioeconomic and environmental factors. The most vulnerable communities have to be identified for the mitigating actions prioritisation within every river basin (United Nations 2009).

Populations in developing countries are more exposed to climate change and they have low capacity to react to the related impacts. There is an urge need to assess vulnerability and to plan adaptation measures. Generally, instead of being proactive communities are not prepared to react to the effects of climate change in West Africa (C3D+ 2013). Recurrent changing climate in West Africa has led to decrease in rainfall since the late 70s (Carbonnel and Hubert 1992; Mahé and Olivry 1995; Nicholson et al. 2000; Mahé et al. 2001; Savane et al. 2001; Tapsoba et al. 2002; Kouakou et al. 2007). This resulted in a reduction of stream flow and wetlands leading to severe droughts (Servat et al. 1998; Madiodio et al. 2004; Kouakou 2011).

Sub-Saharan Africa is mostly vulnerable to the effects of climate change (Boko et al. 2007). Predictions are showing the same impacts. Côte d’Ivoire is also experiencing the negative impacts of climate change (Bigot et al. 2005; Brou 1997; Brou 2005). This has led to decrease in rainfall from 10 to 30 % across the country (Goula et al. 2006, 2009).

People’s perceptions of climate change and the way they respond depend on their knowledge change (Adger et al. 2003; IFAD 2008; Nyanga et al. 2011). Communities know how climate is changing (Heijmans 2001) and their contribution is useful in identifying adaptation strategies.

The aim of the study is to evaluate the vulnerability of different water user groups to climate change and define communities’ adaptation strategies in the Comoe River Basin (CRB). Findings from this study will inform policy development on adaptation measures in the CRB and more widely in West Africa.

## Methods

### Study sites

#### Location and socioeconomic characteristics

Previous studies by Kouakou et al. (2007) as well as Goula et al. (2009) identified the Comoe River Basin (CRB) as one of the most vulnerable basins in West Africa concerning changing rainfall. Also, a diagnostic analysis of the CRB revealed that one of the major problems is changes in the quantity of water and the flows seasonality which has great influence on sustainable development of water supply for industrial and agricultural productions (Goula 2012). The main socioeconomic activities are agriculture, livestock, fisheries, industry, energy, mining, river transport, tourism and crafts.

The CRB is located in West Africa between longitudes 3° and 5°30 West and latitudes 5° and 11°30 North. Comoe is the longest river of Côte d’Ivoire (1160 km). It extends over four countries: two landlocked countries (Burkina Faso and Mali) characterized by a Sudano-Sahelian climate and two coastal countries (Ghana and Côte d’Ivoire) with a tropical and sub-equatorial climate and covers about 78,000 km^2^.

**Fig. 1:**
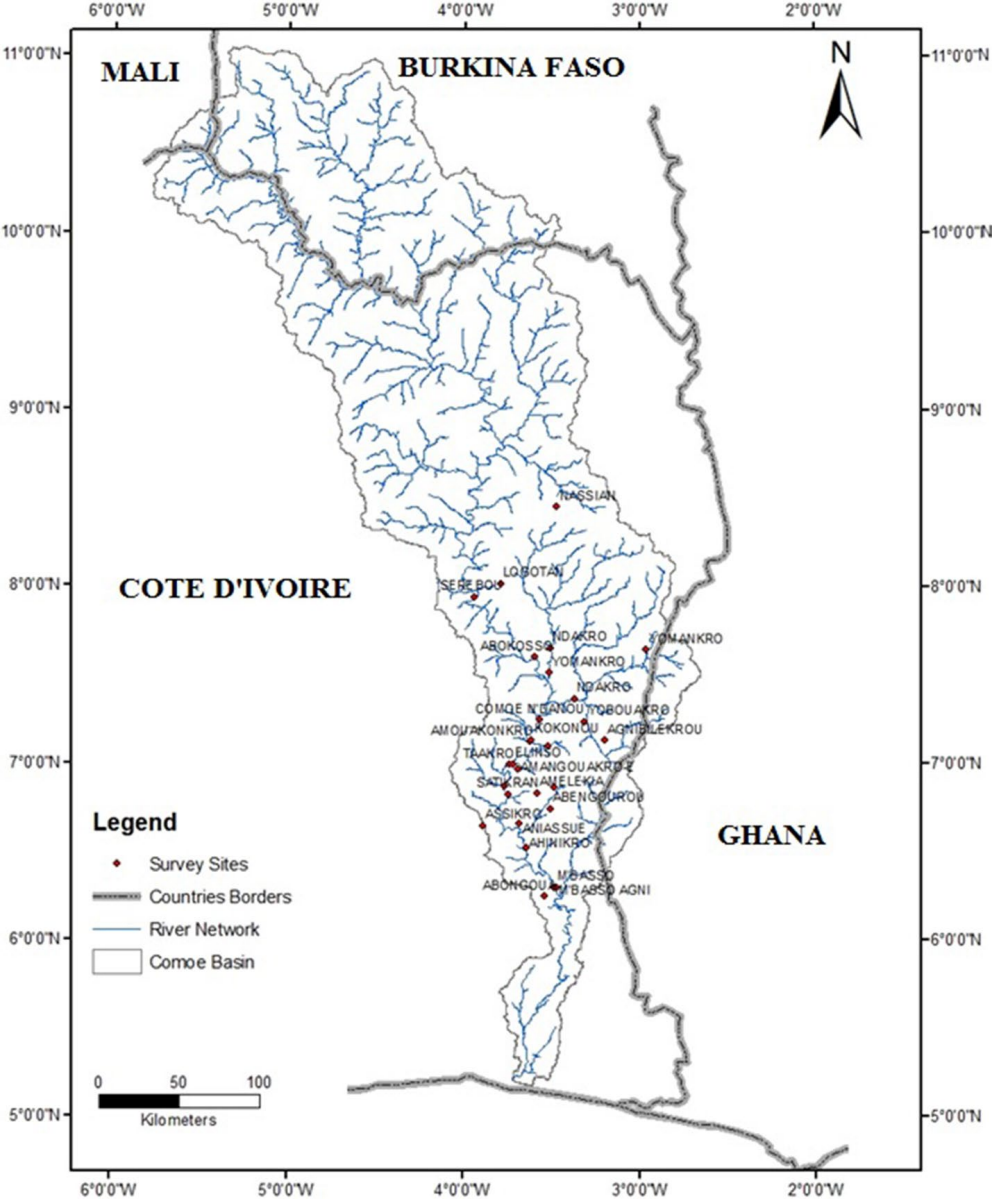
Study sites in the Comoe River Basin

This research was conducted in the southern part of the basin in Côte d’Ivoire. Four (4) regions (Indenie-Djuablin, Me, Gontougo and Iffou) and twenty-one (21) villages along the Comoe River were visited (Fig. 1). Population of the survey areas are multi-ethnic. On the one hand, Attié, Agni, Koulango, Abron, Baoulé and Andoh make up the natives. On the other hand, non-natives are composed of people from Economic Community Of West African States (ECOWAS). Table 1 shows how some key characteristics of the survey area.

**Table 1:** Key demographic and socioeconomic characteristics of the survey area

Characteristics	Study area
Mean annual rainfall (mm)	1100
Rainfall patterns	Bimodal
Mean annual temperature (°C)	28
Main livelihood	Agriculture
Major crops grown	Coffee, Cocoa, Hevea, Cashew, Plantain, Yam, Pepper, Tomato, Gumbo and Aubergine
Types of association (%)	11 % of men, 31 % of women, 28 % of young, 28 % of mutual, 2 % of no association
Population in agriculture (%)	83
Ethnic composition	*Attié, Agni, Koulango, Abron, Baoule* and *Andoh*

According to Salvador et al. (2004), agriculture is still the most important leverage of economic growth in West Africa where the sector plays a significant part in food security. Table 2 shows the percentage of different types of farming in the CRB.

**Table 2:** Type of crops in the study site

Crop type	Crops	Indenie	Me	Gontougo	Iffou
Cash crops	Coffee and cocoa (%)	34	50	25	25
	Cashew nut (%)	34	0	42	50
	Hevea (%)	32	50	33	25
	Total (%)	100	100	100	100
Food crops	Plantain (%)	20	25	13	11
	Yams (%)	20	0	22	22
	Gumbo & Aubergine (%)	20	25	22	22
	Peppers (%)	20	25	22	22
	Tomatoes (%)	20	25	22	22
	Total (%)	100	100	100	100

### Research methods

We assess the droughts in the CRB using the called “Standardized Precipitation Index (SPI) method” elaborated by McKee et al. (1993). Calculation was done with the following formula:

$$SPI_{i} = \left( {\frac{{X_{i} - \bar{X}}}{\sigma }} \right)$$

where $\bar{X}$ indicates the mean and $\sigma$ is the standard deviation of the data series; Negative results from this formula reveal drought events, while positive results show wet events.

The sample size for the survey population was determined using the Cochran (1977) equation.

$$n = \frac{{Z^{2} P\left( {1 - P} \right)}}{{C^{2} }}$$

with n = sample size; Z = confidence level; P = percentage in decimal; c = confidence interval in decimal.

The collection of data used in this study was done during one period of fieldwork; April–May 2014. The survey was conducted with a team of socioeconomics through key informant interviews, questionnaire surveys and Focus Group Discussions (FGDs). This research only considered interviews with water users and stakeholders involved in water use and management.

Information on the perception and the concerns for changing climate and potential adaptation measures and strategies, were collected through two structured questionnaires. The questionnaires were designed in French and the interviews were conducted in French and sometimes in the local languages, *Agni, Baoule, Andoh.* The following headings composed the different sections of the questionnaire:

- Site and respondent: the first section was used to obtain information on the survey site and the water users group.
- Social and cultural information: the second part gives information on the survey site communities, their representativeness, and social organization, everyday diseases of children, men and women.
- Climate context: this part informs on communities’ awareness of changing climate, relative risks, observed effects and local adaptation measures, vulnerable population and economic sectors to changes in climate.
- Livelihood: we used this section to obtain information on communities’ source of income, source of domestic energy, available resources to fight against natural disasters, infrastructures for water management.
- Extreme events: the last section was used to collect information on extreme events frequency, the understanding of these extreme events, the loss of production and reduction of income.

Data from the questionnaires were analysed using Sphinx software. Meteorological data were obtained from the Burkina Faso and Cote d’Ivoire Meteorological Agencies for the period between 1941 and 2010. Rainfall and temperature data showed the trends of these climate variables and how the CRB is vulnerable to climate variability and change.

## Results

Findings are presented in this part by showing the perceptions of changes in climate within the study communities and examining the various adaptation measures used to manage climate change.

### Climate

**Fig. 2:**
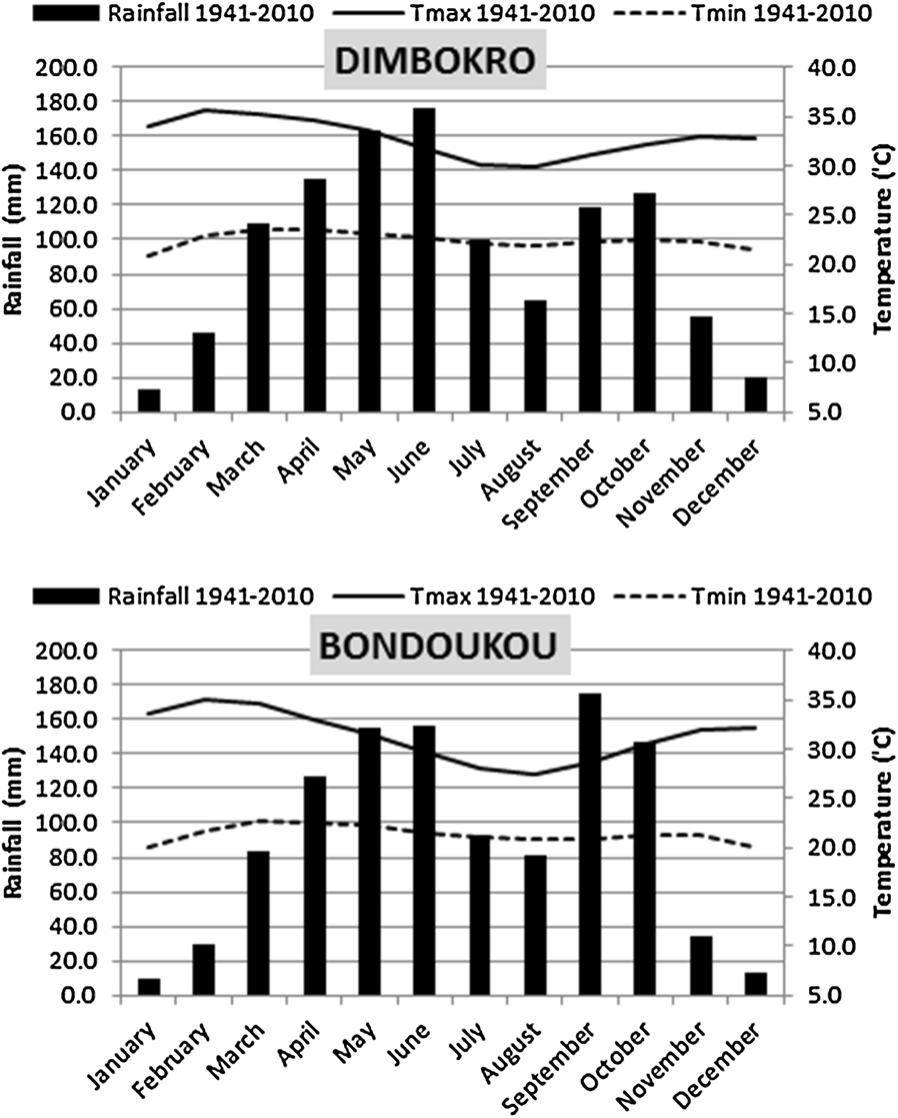
Historical climate monthly averages, Dimbokro and Bondoukou stations, 1941–2010

Crop management needs good knowledge on the timing of the onset and cessation of the rainy season. Also, it is very important to know the period of the year with high rainfall variability (weADAPT 2013). Farming and many sectors are very sensible to rainfall variability. The survey sites lie within the equatorial climate zone (Touchebeuf and Girard 1962; Girard and Sircoulon 1968; Girard et al. 1971). Figure 2 presents historical climate monthly averages for the closest synoptic stations (Dimbokro and Bondoukou) and shows a bimodal rainfall pattern with two rainy seasons for the 1941–2010 period: one is long (from March to July) and another is short (from September to October). June is the wettest month for Dimbokro and September for Bondoukou, with a monthly average around 180 mm from 1941 to 2010. January is the driest month with an average of 13.6 and 9.3 mm of total monthly rainfall respectively for Dimbokro and Bondoukou. The driest period is November throughout February. August corresponds to an intermediary season with low rainfall. The minimum average temperature recorded from 1941 to 2010 is 20.9 and 20 °C in January respectively for Dimbokro and Bondoukou, whereas the maximum average temperature is in February, 35.7 and 35.1 °C.

**Fig. 3:**
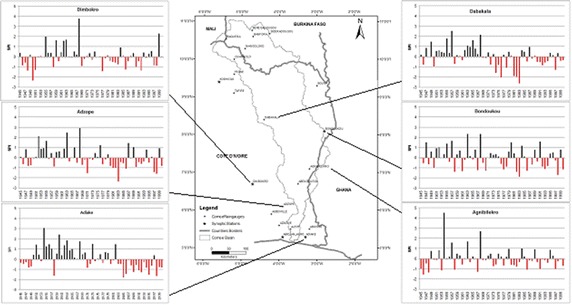
Interannual variability of rainfall in the study sites, 1945–2000

A 55-years record of data from 6 rain gauges of the survey area examined for temporal distribution show a succession of humid and drought periods with an extreme dry period beginning in 1969 (Fig. 3). Table 3 shows a delay and a shortage of rainy seasons at Abidjan, Adiake and Dimbokro stations.

**Table 3:** Delay in rainy seasons

Station	Period	First rainy season			Second rainy season		
		Start	End	Length (days)	Start	End	Length (days)
Abidjan	1951–1980	Mar-27	Aug-22	149	Oct-11	Dec-31	82
	1971–2000	Apr-03	Aug-17	137	Oct-17	Dec-31	76
	Delay (days)	−7	−5	−12	−6	0	−6
Adiake	1951–1980	Mar-21	Sep-23	187	Sep-28	Dec-30	93
	1971–2000	Apr-08	Sep-14	160	Sep-30	Dec-24	85
	Delay (days)	−18	−9	−27	−2	−6	−8
Dimbokro	1951–1980	Apr-03	Jul-29	117	Sep-11	Nov-03	53
	1971–2000	Apr-04	Jul-11	98	Sep-06	Nov-08	63
	Delay (days)	−1	−18	−19	5	5	10

**Fig. 4:**
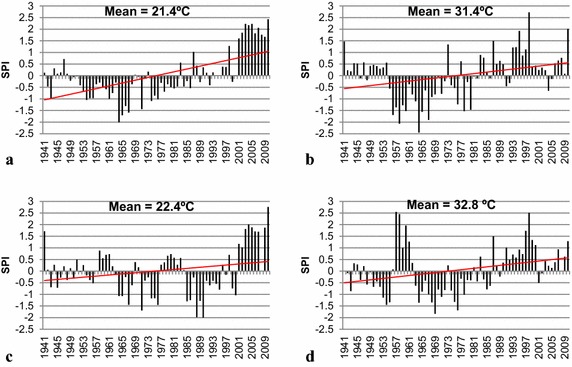
Interannual variability of **a** minima temperature at Bondoukou, **b** maxima temperature at Bondoukou, **c** minima temperature at Dimbokro, **d** maxima temperature at Dimbokro, 1941–2010

The interannual variability of temperature for the period 1941–2010 at Bondoukou and Dimbokro synoptic stations shows a continuous increase of temperature (Fig. 4). This increase is more pronounced at the minima with about 2 °C than maxima level with about 1 °C. The average minima temperatures are 21.4 and 22.4 °C while the average maxima temperatures are 31.4 and 32.8 °C respectively at Bondoukou and Dimbokro. Around 1975, the observed interannual values exceeded the 1941–2010 interannual mean.

Temperature projections over West Africa using CMIP5 GCMs for RCP4.5 and RCP8.5 (Meehl et al. 2007; Fontaine et al. 2011; Diallo et al. 2012; Monerie et al. 2012) and regional downscaling (Patricola and Cook 2010, 2011; Mariotti et al. 2011; Vizy et al. 2013) are indicating an increase from 3 to 6 °C by the end of this century. Because of convective rainfall in this region, precipitation projections are showing inter-model variation in both the amplitude and direction of change (Niang et al. 2014; Biasutti et al. 2008; Druyan 2011; Fontaine et al. 2011; Roehrig et al. 2013). For the Comoe River Basin, climate projections for this century (2031–2040 and 2094–2100), Regional Climate Model version 3 is indicating a decrease in average total monthly rainfall by 10 and 20 % for the whole basin and a clear increase in monthly average temperature, about 0.62–0.74 °C for the period 2031–2040 and 3–4.1 °C for the period 2094–2100 (Kouakou 2011).

### River discharge

**Fig. 5:**
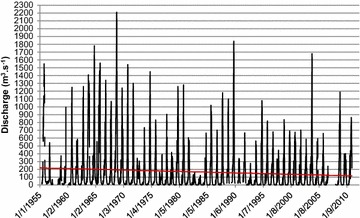
Discharge trend in the Comoe River at M’Basso gauging station, 1955–2010

A 55-years record of river discharge at M’Basso gauging station where the mean annual flow is 167.5 m^3^ s^−1^, shows a decline of discharge of about 100 m^3^ s^−1^ in the Comoe River (Fig. 5).

### Water users’ awareness of climate change

**Fig. 6:**
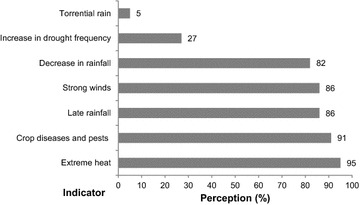
Water users’ perceptions about climate change in the survey area, n = 384

The questionnaire survey was used to know if water users have heard of climate change before or if they have never heard of this concept. 95 % of the interviewee in the study communities had heard of it and are aware that climate change is occurring. They had perceived climate change in terms of various changes (Fig. 6). Among them, 86 % have observed late rain. Another 82 % of the opinion was that rainfall has decreased and therefore increased the frequent occurrence of droughts (27 %). Majority of the water users (95 and 86 %) interviewed believe that temperature and winds are becoming warmer and stronger respectively. Going by the opinion of 91 % of water users, the crop diseases and pests have increased. Only 5 % of them were observing torrential rain. There was almost unanimous agreement across the water users that there is a delay in the onset of the rainfall compared with their childhoods.

The answer of the origin of climate change varies from literate to illiterate people. Literate interviewed water users define climate change as a change in, for example, rainfall and temperature from the past to now. For the illiterate, climate change is a divine sanction against human who have broken social values. They were of the opinion that rainfall is not coming as more as in the past because of deforestation.

### Socioeconomics effects of climate change

**Fig. 7:**
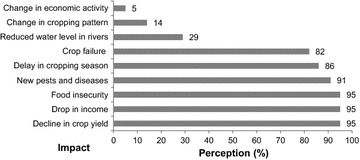
Perceived impacts of climate change in the survey area, n = 384

Agriculture is the largest economic sector in the CRB and most communities reported that they have been experiencing changes in climate (Fig. 7). According to 95 % of the interviewed people, crop yield is decreasing over years because of climate change, ultimately leading to drop in income and food insecurity. About 91 % of water users noticed new pests and diseases like *Swollen*-*Shoot* in cocoa and malaria for human. While 86 % of interviewed people reported a delay in cropping season, 82 % noticed crop failure. One-third of them have perceived declining water level in rivers. According to 14 % of the water users, there is a change in cropping pattern. About 5 % of the interviewed people reported a change in economic activity. It was the case of the fishermen community of M’Basso called ‘Bozos’ who became farmers.

### Local adaptation measures

**Fig. 8:**
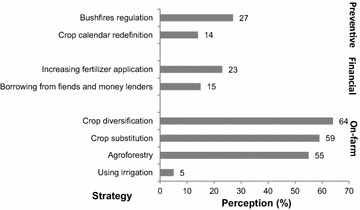
Adaptation strategies practiced by study communities, n = 384

To manage the changes perceived by communities in the CRB, various adaptation strategies have been employed. Figure 8 summarizes these and shows three broad strategies, namely, on-farm, financial and preventive adaptation strategies are undertaken by interviewed people in response to the effects of climate change.

Firstly, on-farm adaptation measures refer to agricultural management practices undertaken in the study communities and include crop diversification (64 %), crop substitution (59 %), agroforestry (55 %) and irrigation (5 %). Secondly, financial adaptation strategies refer to income management strategies to sustain livelihood during climate adversities. These strategies include increasing fertilizer application (23 %) to improve crop productivity and borrowing from friends and money lenders (15 %). Thirdly, preventive adaptation strategies that were reported include bushfires regulation (27 %) and crop calendar redefinition (14 %).

## Discussion

According to many authors, land-cover changes in tropical and subtropical Africa have led to accentuate drought occurring (Zeng and Neelin 2000; Pielke 2001; Semazzi and Song 2001; Zeng et al. 2002; Madiodio et al. 2004; Paeth et al. 2009). Both the decline of rainfall and the clear temperature rising by the middle and end of the century were found to conform to others climate projections (IPCC 2012; ECOWAS-SWAC 2008).

Water users in the CRB have heard of climate change and their understanding of it is closed to scientist definition: climate change is a change in, for example, rainfall and temperature from the past to now. Most water users have noticed that temperatures are getting warmer and there is a decline in rainfall over the last three decades. These perceptions were found to conform to the perceptions of water users in Cote d’Ivoire the last forty years (Bigot et al. 2005; Brou 2005) and in many African countries (Madison 2007; Nhemachena and Hassan 2007; Gbetibouo 2009; Ishaya and Abaje 2008; Dinar et al. 2008; Lema and Majula 2009; Mertz et al. 2009; Ayanwuyi et al. 2010; Mensah et al. 2010; Mubaya et al. 2010; Akponikpe et al. 2010; Acquah 2011; Kemausuor et al. 2011; Bryan et al. 2011; Deressa et al. 2011). Findings from Nicholson (2001) and Hope (2011) have showed that temperatures vary from day to night and throughout the year in tropical areas. Water users were also of the opinion that deforestation for cropping contribute to changes in climate.

One of the key perceived effects of changes in climate was declining in crop yield. Most of cash crops need good rainfall and their development is done to the detriment of forest areas. According to Brou (2005), Ohouo and Obodi (2011) the rate of deforestation is one of the most important rates in the world (90 %). It is the case of cocoa which need annual rainfall between 1200 and 1500 mm (Brou et al. 2003). Any change in such condition lead to declining in crop yield (Surre and Coomans 1972; Yao et al. 1995; Yao and Kamagate 2010). One net impact of climate change reported by respondents was pest and diseases. The recommendations for the described Cocoa swollen-shoot virus are to cut down alternative hosts. This results in a decrease of cocoa yield (Dzahini-Obiatey et al. 2010; Domfeh et al. 2011). The presence of mosquitoes is mainly a question of temperatures and humidity. Increased temperatures will increase occurrence of malaria. Crops destruction is increasing the cost of food because it is leading to low yields, food shortage and communities incomes declining (MICCA 2012). Food insecurity due to climate change is reality in the CRB as well as in various geographic areas of Cote d’Ivoire (Yabile 2013).

Various adaptation measures are employed by water users to respond to climate change. The foremost adaptation strategy adopted by the study communities is crop diversification. The advantage of crop diversification is in the compensation of one crop failure by the yield of another crop. Many crops are grown at the same time on the same field. Association used for cash food are cocoa + plantain; Cocoa + cashew nut; Cocoa + cashew nut + teak. For food crops concern mainly yams, groundnut, taro, tomatoes, pepper and aubergine. The second important strategy is crop substitution. Water users are now using drought-tolerant crops to secure their occupation and income from the adversities caused by climate change. Thus, they have been replacing cocoa and coffee by hevea, cashew nut and teak. Also, long duration crops and varieties are getting replaced by short duration ones. For example, the variety of cocoa called ‘French cocoa’ has been replaced by ‘Mercedes cocoa’ promoted by ‘*Centre National de Recherche Agronomique (CNRA)*’. This adaptation strategy has a negative consequence the region is nomore called ‘*boucle du cacao*’ and people are immigrating to the South East of the country. Another adaptation strategy used by the study communities is agroforestry. It concerns the use of fast-growing-species of trees in the farm. The frequent tree species are teak (*Verbenaceae*), framire (*Terminalia ivorensis*) and frake (*Terminalia superba*). A respondent states for example ‘when there is forest, there is rain and wind speed is reduced’. Bushfires and crop calendars redefinition are preventive adaptation strategies used by water users. Respondents reported that bushfires are now well regulated with the actions of local comities and non-governmental organizations (NGO) like « *SIN NAN SOPKA MIN* » (e.g. fire made me poor). Also, crop calendars are redefined to facilitate adaptation by way of planning when to plant their crops. Time of rainfall has changed over the years, what the respondents termed as ‘untimely rainfall’ that poses much difficulty in the cultivation of crops. They usually manipulate the sowing date in accordance with the arrival of rain. Some water users rely on past rainfall patterns including the start and ending of the rainy season to form expectations and predict the rainfall patterns for the coming season. This redefinition of seasonal calendars is done most of the time in collaboration with some national agencies like ANADER (*Agence National d’Appui au Développement Rural*), FIRCA (*Fonds Interprofessionnel pour la Recherche et le Conseil Agricoles*) and SAPH (*Société Africaine de Plantations d’Hévéa*). Increasing fertilizer application and borrowing from friends and money lenders are financial adaptation strategies used by water users in the CRB. They resorted to these strategies to keep the household food secure and sometimes after they have run out of provisions from their own production. Getting money enables farmers to look for fertilizer and seeds of improved varieties.

## Conclusions

This study presented water users’ perception on changing climate in the study site based on interviews and questionnaires. Findings have revealed that 95 % of the sample in the study communities had heard of climate change and are aware that it is happening. This study also, found that agriculture is the largest economic sector in the CRB. Communities have experienced changing climate as changes in economic activity and cropping pattern, reduced water level in rivers, crop failure, delay in cropping season, new pests and diseases, food insecurity, drop in income and decline in crop yield. Others results revealed that communities have employed various adaptation strategies such as crops diversification, substitution and calendar redefinition, agroforestry, borrowing from friends and money lenders, increasing fertilizer application, bushfires regulation and irrigation.

## Abbreviations

ANADER: Agence National d’Appui au Développement Rural
BMBF: German Federal Ministry of Education and Research
C3D+: Climate Change Capacity Development
CCPAN: Climate Change Philanthropy Action Network
CMIP5: Coupled Model Intercomparison Project Phase 5
CNRA: Centre National de Recherche Agronomique
CRB: Comoe River Basin
ECOWAS-SWAC: Economic Community of West African States-Sahel and West Africa Club
FIRCA: Fonds Interprofessionnel pour la Recherche et le Conseil Agricoles
FGDs: Focus Group Discussions
GCMs: Global Climate Models
GRP: graduate research program
IFAD: International Fund for Agricultural Development
IPCC: intergovernmental panel on climate change
MICCA: mitigation of climate change in agriculture
NGO: non-governmental organizations
RCP4.5 and RCP8.5: representative concentration pathways 4.5 and 8.5
SAPH: Société Africaine de Plantations d’Hévéa
SPI: Standardized Precipitation Index
WASCAL: West African science service center on climate change and adapted land use
